# Effect of Coadministration of Neurovite and Lamivudine on the Histomorphology of the Cerebellum of Wistar Rats

**DOI:** 10.1155/2014/258040

**Published:** 2014-01-16

**Authors:** A. I. Peter, M. B. Ekong, K. Davies, O. O. Azu, R. B. Bassey, L. O. Ugwu, I. U. Umoh

**Affiliations:** ^1^Department of Anatomy, Faculty of Basic Medical Sciences, University of Uyo, PMB 1017, Uyo, Nigeria; ^2^Department of Physiology, Faculty of Basic Medical Sciences, University of Uyo, PMB 1017, Uyo, Nigeria; ^3^Discipline of Clinical Anatomy, School of Laboratory Medicine & Medical Sciences, Nelson R Mandela School of Medicine, Private Bag X54001, Congella 4013, Durban, South Africa

## Abstract

*Introduction*. Lamivudine is a nucleoside reverse transcriptase inhibitor antiretroviral agent used in the treatment of human immunodeficiency virus type 1 infection. This study was to investigate the effects of coadministration of neurovite and lamivudine on the histomorphology of the cerebellum of Wistar rats. *Materials and Methods*. Twenty Wistar rats were divided equally into four groups. Group A animals were the control treated with distilled water. Groups B, C, and D animals were treated, respectively, with therapeutic dose of lamivudine (4.28 mg/kg), a combination of lamivudine (4.28 mg/kg) and neurovite (7.05 mg/kg), and neurovite (7.05 mg/kg) alone, daily. The rats were sacrificed using chloroform inhalation, processed, and stained using H&E method. *Results*. There was severe cellular degeneration with dystrophic changes, vacuolization in the molecular and granular layers, and aggregation of swollen Purkinje cells in group B animals compared with group C animals which showed only slight cellular dystrophy and inflammation. The mean cellular population was significantly (*P* < 0.05) higher in the treatment groups compared with the control. *Conclusion*. There was amelioration of damage of the cerebellum in the animals treated with neurovite and lamivudine combination compared to animals treated with only lamivudine. Therefore, there is need to give neurovite to patients on lamivudine therapy.

## 1. Introduction

Lamivudine (INN)^6^ or 3TC is a levorotary pyrimidone-1,3-oxathiolane derivative and has the molecular formula C_8_H_11_N_3_O_3_S. Lamivudine is an effective and well-tolerated agent for treating chronic hepatitis B infection and acquired immunodeficiency syndrome [1, 2]. It is an antiretroviral drug in the therapeutic category of nucleoside reverse transcriptase inhibitor [2]. Lamivudine is very useful in preventing HIV and hepatitis B from multiplying by way of its active form, lamivudine triphosphate (3TCTP) which is generated via intracellular triple phosphorylation process. Lamivudine competitively inhibits viral transcriptase by causing termination of DNA replication, thus interrupting HIV replication [3].

Antiretroviral treatment can significantly prolong the lives of people living with HIV. Modern combination therapy is highly effective and people with HIV and on antiretroviral treatment could live for the rest of their lives without developing AIDS [4]. Despite these improvements, the prolonged use of highly active antiretroviral therapy (HAART) has led to certain neurologic complications such as myelopathy, neuropathy, neuropathic pain, and cognitive decline [5, 6].

There is a report that even low concentrations of antiretroviral (ARV) drugs that penetrate the blood brain barrier have detrimental effects on the central nervous system [7]. Other toxicities induced by anti-HIV drugs include hepatotoxicity, allergies, hyperglycaemia, lactic acidosis, lipodystrophy, and gastrointestinal disorder [8]. Cognitive impairment occurs in a substantial (15–50%) proportion of HIV-infected patient on highly active antiretroviral therapy (HAART). [9–11] It has also been reported that about 40% of patients treated with lamivudine develop toxicities related to the central nervous system, with symptoms such as dizziness, insomnia, and depersonalization [12].

Neurovite on the other hand is the pharmaceutical grade supplements that provide superior nutritional support for the brain and body. It is a brain-directed broad spectrum of daily multinutrient. The constituents of neurovite include thiamine (vitamin B1), B6, and B12. Vitamin B1 serves as a cofactor for several enzymes involved in energy. Vitamin B6 (pyridoxine) is a water-soluble vitamin that is essential for cellular function and growth due to its involvement in important metabolic reaction (Vitamins-Supplements Org). Cyanocobalamin (vitamin B12) plays a role in the conversion of homocystidine to methionine, with CoA and Krebs cycle being also dependant on B12. Vitamin B12 helps nerve cell and red blood cell and in manufacturing and repairing DNA, as well as assisting in memory [13].

Vitamin B deficiencies have been well documented in HIV-positive patients even in early asymptomatic states. Beach et al. [14] reported low levels of B6, B12, and riboflavin in 53%, 23%, and 26%, respectively, in patients. The aim of this research therefore was to study the effects of coadministration of neurovite and lamivudine on histomorphology of the cerebellum of treated Wistar rats.

## 2. Materials and Methods

Twenty Wistar rats were obtained from the Animal House of the Faculty of Basic Medical Sciences, University of Uyo. The rats were divided into four groups of five animals each. Group A animals were the control and were treated with 1 mL of distilled water. Group B animals were treated with the therapeutic dose of lamivudine (4.28 mg/kg) daily, and group C animals were treated with a combination of the therapeutic doses of lamivudine (4.28 mg/kg) and neurovite (7.05 mg/kg) daily, while group D was treated with only neurovite (7.05 mg/kg) daily.

The animals were handled according to the guidelines for the treatment of laboratory animals. The rats were treated for 28 days and allowed water and feed *ad libitum*. On the 29th day, the rats were sacrificed using chloroform inhalation method. The cerebella (neocerebellum) were excised, routinely processed, stained using haematoxylin and eosin method, and viewed under light microscope.

The cellular population, size, and area covered were determined with the ImageJ software. Statistical analysis was applied to determine the difference using one-way analysis of variance (ANOVA), and a post hoc Tukey's test was applied. All data were considered significant at *P* < 0.05.

## 3. Results

The histomorphological features that are present in the various groups upon viewing under the light microscope are as follows.

**Figure 1:**
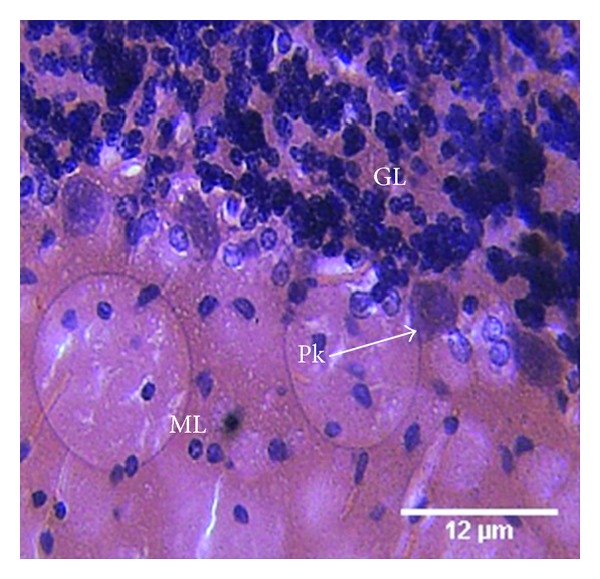
Photomicrograph of the histology of the cerebellum of the control group (A) shows the three cerebellar cortical layers: molecular (M) layer, granular (GL) layer, and the single-line Purkinje (P) cells. Within the molecular layer, the basket and stellate neuronal cells are well shown. No apparent abnormality is seen. H & E, ×400.

The control group (A) was administered 1 mL of distilled water. Here the photomicrograph of the histology of the cerebellum showed the three cerebella cortical layers: molecular (M) layer, granular (GL) layer, and the lining Purkinje (P) cells. Within the molecular layer the basket and stellate neuronal cells are well shown. No apparent abnormality is seen (Figure 1).

**Figure 2:**
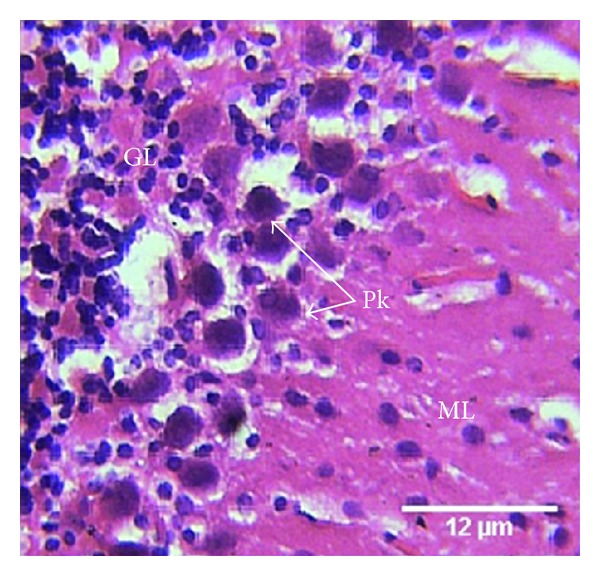
Photomicrograph of the histology of the cerebellum of group B treated animals treated with lamivudine (4.28 mg/kg) shows the three cerebellar cortical areas: molecular (ML) layer, granular (GL) layer, and disrupted and swollen Purkinje (Pk) cells. The Purkinje cells appear as aggregates, with disrupted white matrix. The granular cells appear scattered. H & E, ×400.

Group B treated with 4.28 mg/kg of lamivudine showed the histology of the cerebellum of group B animals with aggregated, swollen Purkinje cells, and disrupted white matrix. The granular cells appear scattered (Figure 2).

**Figure 3:**
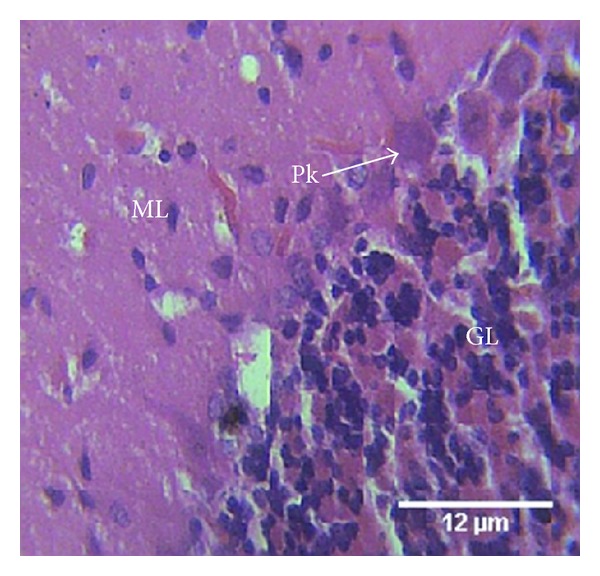
Photomicrograph of the histology of the cerebellum of group C animals treated with 4.28 mg/kg of lamivudine shows the three cerebellar cortical areas: molecular (ML) layer, granular (GL) layer, and single-cell layer of Purkinje (Pk) cells. There is slight aggregation of the granular cells, with slight disrupted matrix. H & E, ×400.

Group C treated with a combination of the therapeutic doses of lamivudine (4.28 mg/kg) and neurovite (7.05 mg/kg) showed slight aggregation of the granular cells, with slight disrupted matrix (Figure 3).

**Figure 4:**
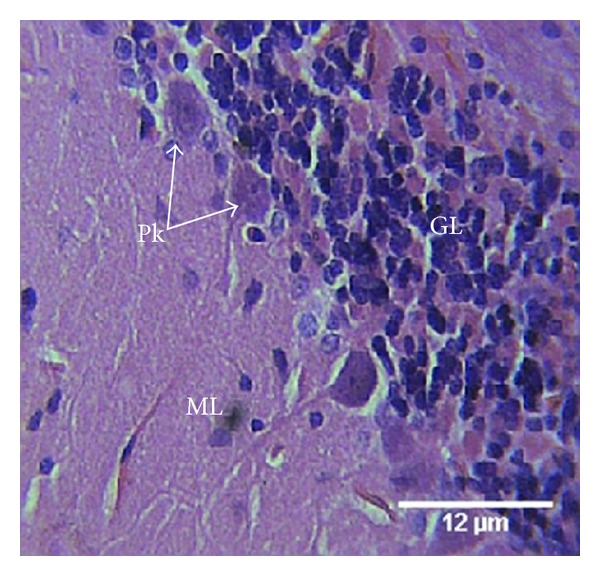
Photomicrograph of the histology of the cerebellum of group D animals treated with 7.05 mg/kg of neurovite shows the three cerebellar cortical areas: molecular (ML) layer, granular (GL) layer, and Purkinje (Pk) cells. The granular cells aggregate, and the Purkinje cells are single-lined. H & E, ×400.

Group D treated with 7.05 mg/kg of neurovite showed the granular layer with cells being aggregated. The Purkinje cells were arranged singly and in line (Figure 4).

The mean cellular population in the treated groups B, C, and D was significantly (*P* < 0.05) higher compared with the control. However, the group treated with neurovite only had significant (*P* < 0.05) higher cellular population than groups B and C treated, respectively, with lamivudine only, and, lamivudine and neurovite combination, while group B had a significant (*P* < 0.05) lower cellular population compared to group C. Mean cellular size and total cellular area in the treated groups were however not different from the control (Table 1).

## 4. Discussion

Cerebellum is known as a motor control centre, and it is increasingly recognized as contributing to general cognitive processing and emotional control [15–17]. However, findings have shown that the cerebellum may be able to perform cognitive activities independent of motor function [18, 19]. Studies on rats have associated HIV infection with increased neuronal degeneration and death [20, 21]. But little is known concerning the extent of cerebella atrophy in HIV patients due to the effect of the drugs and how it relates to cognition.

In this study, the group treated with only lamivudine showed swollen Purkinje cells, loss of granular cell aggregation, and disruption of brain matrix, with higher cellular population. This may be due to over excitation of the neurons, and gliosis, as well as depletion of myelin. These conditions may lead to cytotoxic degeneration of the neuronal cells [22]. These results may be one reason that postmortem studies of HIV-positive patients show cerebella degeneration, diffuse loss of neurons in the granule cell layer, white matter degeneration, and cerebella atrophy [23].

The cerebellum of group C animals treated with a combination of lamivudine and neurovite showed slight effect of myelin loss but higher cellular population, an indication of gliosis. The changes seen in this group may be attributed to lamivudine, which may have been modulated by neurovite. HIV-infected patients require levels of B-vitamins in multiples of the daily requirement in order to achieve normal plasma levels. Low plasma level of vitamins B12 and B6 appears to be widespread in HIV infection. There are reports that significant decreases in risk of progression to AIDS were observed with B-vitamin intake [24, 25]. These reports support our results as the toxic effect of lamivudine was less apparent when combined with neurovite. The cerebellum of group D animals treated with neurovite only showed normal cells and layers of the cerebellum, an indication that neurovite may be a brain health booster.

In conclusion, lamivudine treatment may cause histopathological changes in the cerebellum, which may be ameliorated by neurovite. Hence, neurovite is recommended for coadministration to patients on lamivudine therapy.

## Figures and Tables

**Table 1:** Cellular population, size, and area of the treated and control groups.

Group	Mean cellular population	Mean cellular size (*μ*m^2^)	Total cellular area (*μ*m^2^)
Control (A)	355 ± 1.581	2 ± 0.173	650 ± 10.000
Group B	386 ± 3.421*	1 ± 0.063^NS^	350 ± 7.071^NS^
Group C	413 ± 1.140^∗,b^	1 ± 0.119^NS^	371 ± 3.421^NS^
Group D	453 ± 1.703^∗,b,c^	1 ± 0.161^NS^	510 ± 1.613^NS^

*Significantly higher than group A at *P* < 0.05.

^NS^Not Significantly higher than group A at *P* < 0.05.

^b^Significantly higher than group B at *P* < 0.05.

^c^Significantly higher than group C at *P* < 0.05.
